# Structural Racism and Cancer: Calls to Action for Cancer Researchers to Address Racial/Ethnic Cancer Inequity in the United States

**DOI:** 10.1158/1055-9965.EPI-21-1179

**Published:** 2022-06-01

**Authors:** Alicia L. Best, Mya L. Roberson, Jesse J. Plascak, Caryn E. Peterson, Charles R. Rogers, Theresa A. Hastert, Yamilé Molina

**Affiliations:** 1 College of Public Health, University of South Florida, Tampa, Florida.; 2 Department of Health Policy, Vanderbilt University School of Medicine, Nashville, Tennessee.; 3 Division of Cancer Prevention and Control, The Ohio State University College of Medicine, Columbus, Ohio.; 4 Division of Epidemiology and Biostatistics, University of Illinois at Chicago School of Public Health, Chicago, Illinois.; 5 Department of Family & Preventive Medicine, University of Utah School of Medicine, Salt Lake City, Utah.; 6 Karmanos Cancer Institute, Wayne State University School of Medicine, Detroit, Michigan.; 7 Division of Community Health Sciences, University of Illinois at Chicago School of Public Health, Chicago, Illinois.

## Abstract

As leaders with the American Society of Preventive Oncology (ASPO) Cancer Health Disparities Special Interest Group, we describe the role of structural racism in perpetuating cancer health inequity historically, and potential implications of COVID-19 in exacerbating the effects of structural racism on patients with cancer seeking screening, diagnostic care, treatment, and survivorship support. As a strategy to reduce cancer inequities in the United States, we provide the following calls to action for cancer researchers to help alleviate the burden of structural racism: (i) identify and name structural racism while describing its operation within all aspects of scientific research; (ii) comprehensively integrate discussions on structural racism into teaching, mentoring, and service activities; and (iii) understand and support community actions to address structural racism.

Cancer remains the second leading cause of death in the United States, and cancer-related health disparities are well documented in the scientific literature (1). The American Society of Preventive Oncology (ASPO), a multidisciplinary society committed to advocating for cancer prevention and control research through professional education and development opportunities, has demonstrated commitment to cancer health equity primarily through: (i) highlighting cancer health disparities at annual meetings (e.g., poster/oral presentations, symposia, etc.); and (ii) establishing the Cancer Health Disparities Special Interest Group, which has resulted in multifaceted activities centered on promoting cancer health inequities-focused research outside of annual meetings (e.g., webinars, newsletters, social media). Recently, ASPO made significant strides to promote research specifically focused on structural and contextual determinants of cancer inequity. This commitment to cancer health equity is exemplified by symposia topics chosen for the 2021 annual meeting, including unequal access as a multidimensional public health problem, and structural racism, as well as a recent public commitment to anti-racism (ASPO Commitment to Anti-Racism - American Society of Preventive Oncology).

## Structural Racism and Cancer Disparities

Abundant evidence supports the relationship between societal factors and health outcomes, including those related to cancer. Many observed racial/ethnic disparities in cancer outcomes are avoidable and thus, unjust (2). Structural racism, which contributes to many health disparities, involves the complex interplay of macro-level systems, policies, as well as institutional practices and processes which accumulate over time and result in the unfair advantage of one racial group over another (3). In the United States, structural racism (distinguished from racial categorization) manifests as advantages to people who are racialized as White and disadvantages primarily to people who are racialized as Black/African American, Asian, people indigenous to North America, and individuals of Hispanic/Latino/Latinx ethnicity [hereafter, BIPOC (Black, Indigenous, and other People of Color); ref. 3]. These broad categories included in our present conceptualization of BIPOC reflect those most disproportionately burdened by negative cancer- and COVID-19–related outcomes. These categories are not homogenous and encompass a variety of ethnic groups and diverse experiences. Importantly, structural racism functions through societal power (e.g., economic, political, social) – including unequal distributions of resources and hazards as well as socioculturally constructed justifications for inequality (e.g., BIPOC as weak/unable to claim the promise of resources or aggressive/undeserving of resources). These processes go beyond the beliefs or behaviors of individuals, and efforts to address individual racial prejudice or intentional racial discrimination fail to address the structural dynamics that contribute to health disparities. Moreover, by its nature, structural racism is embedded within American society and can be difficult to identify and/or inappropriately dismissed; thus, the effects of structural racism are often attributed to BIPOC’s culture and/or individual deficits rather than the imbalance of societal power. Identifying and understanding ways in which structural racism may contribute to established racial disparities in cancer outcomes is critical to reducing its impact and improving outcomes for BIPOC.

## COVID-19 and Cancer Disparities

The impacts of structural racism on cancer disparities may be amplified by other co-occurring crises, including the COVID-19 pandemic. BIPOC communities have been disproportionately impacted by COVID-19, including experiencing higher rates of infection, serious illness, and death, while Asian populations, for example, have experienced racism at interpersonal *and* structural levels in relation to the COVID-19 pandemic (4). Individual experiences with COVID-19, high community rates of infection, and efforts to mitigate community spread (4) could potentially impede patients’ ability to access cancer care. This is particularly detrimental to BIPOC who are more likely to be diagnosed with cancer at later stages and experience delays in initiating treatment prior to COVID-19. Moreover, many cancer survivors rely on multigenerational family and community support networks for transportation to and from appointments; delivery of food, medicine, and necessary supplies; and provision of social and emotional support. Disruptions to these social support networks due to COVID-19 is especially burdensome for cancer survivors. Finally, other systemic factors not directly related to healthcare access may also exacerbate the impact of structural racism on cancer inequities during the era of COVID-19. For example, many BIPOC communities also experience other social issues (e.g., food insecurity, lack of transportation and continuous access to utilities and stable housing) that compound cancer burden, as well as lead to worse COVID-19 outcomes.

## Calls to Action for Cancer Researchers

By its nature, structural racism is endemic in our society; and coinciding public health emergencies such as the COVID-19 pandemic magnify its negative effect on cancer outcomes. As such, individual and organizational influence can and must be used to begin dismantling these systems of injustice to eliminate cancer inequities. Below we offer suggestions for ways in which cancer researchers can address structural racism and support BIPOC communities in their: (i) scholarship and process of scientific inquiry; (ii) teaching, mentorship, and service within their institutions; and (iii) community engagement.

## Call to Action #1: Explicitly Name Structural Racism and Describe Its Operation in Scientific Research

The cancer research community must take steps to become anti-racist within all aspects of scientific conduct, including approaches to scientific inquiry. As an important first step, we need to recognize that cancer research of any type—basic, clinical, behavioral, psychosocial, etc.—operates within and is affected by structural racism. Beyond cataloging cancer disparities and describing the different, often worse, outcomes in BIPOC communities, we must explicitly identify ways in which societal forces, including structural racism, contribute to those outcomes. Next, to achieve anti-racist scientific inquiry, cancer disparities researchers should explicitly name and identify the role of structural racism in cancer inequities. These actions can help prevent its perpetuation and reproduction within research and society.

Investigators engaging in observational and intervention cancer research should consider how *past and present* structural racism influences study elements (e.g., study team diversity/inclusivity; protocol cultural acceptance/appropriateness; barriers to participation for BIPOC; causal relationships with exposure/outcome; dissemination/implementation; etc.). Use of theoretical/conceptual frameworks that account for structural racism (e.g., Critical Race Theory, Social Determinants of Health, Fundamental Cause Theory) should influence all facets of cancer disparities research. Other suggested strategies to address structural racism in cancer disparities research include: (i) actively seeking, continually engaging, and appropriately compensating BIPOC study team members at all levels (e.g., investigators, research assistants, community stakeholders); (ii) measuring and estimating effects of structural racism within studies (e.g., residential segregation; mortgage lending discrimination; land use policy; police practices; targeted marketing); (iii) naming racism in research dissemination (e.g., distinguishing between the role of ancestry/genetics and structural racism in peer-reviewed publications); and (iv) addressing structural racism in implementation efforts. For example, cancer disparities interventions that employ strategies such as implicit bias and/or empathy training may address personally mediated racism; however, these individual-level approaches alone do not acknowledge or address the role of power imbalances and thus, are not sufficient to address structural racism.

## Call to Action #2: Integrate Discussions on Structural Racism into Teaching, Mentoring, and Service Activities

Beyond scholarship, cancer disparities researchers must use our positions of power to address historical racial inequities in tangible ways through teaching, training, mentoring, and advocacy for students and scientists who identify as BIPOC. Where appropriate, required and elective courses, and other trainings should include accurate accounts of relevant history and explicitly address the role of structural racism in creating bias in health-related and other scholarship. Such content should seek to eradicate larger societal narratives focused on individual blame, and instead, emphasize the fundamental role of power, societal responsibility, *and* individual/community agency. For instance, re-education about the history of race and racism in the US is necessary to counter inaccurate distinctions between populations racialized as Black, African American, and other BIPOC groups. These categories are socially constructed and not mutually exclusive, and their continued use masks the true magnitude of anti-BIPOC racism. In addition, instructors should showcase the writing and research of BIPOC scholars and integrate this work into course content. Instructors should also advocate for funding, scholarships, and assistantships that provide adequate economic support to eliminate the economic barriers faced by many BIPOC individuals pursuing graduate education. Finally, instructors should advocate for structural competency training and other pedagogic opportunities to equip cancer researchers and other professionals with the knowledge and skills necessary to recognize and address structural racism in their research programs, within their institutions, and across systems.

Regarding research training and mentorship, cancer disparities researchers must commit to building teams that include representation from BIPOC communities. Similarly, mentorship must support personal and academic development in ways that both acknowledge power differentials and other contextual realities of BIPOC mentees and support their potential. Researchers can support BIPOC mentees through the provision of high-quality research and mentorship opportunities, including opportunities to contribute to and/or lead peer-reviewed publications and conference presentations; providing strong letters of support for training and employment applications; proactively helping to connect mentees with cancer and other relevant scholars; and publicly promoting mentee successes and accomplishments.

Outside of their own research positions, cancer disparities researchers can promote BIPOC student success through dissemination of information about training opportunities to BIPOC students and trainees throughout their institutions. For example, cancer researchers can pursue training grants which support BIPOC scholars, encourage and endorse applications for funding mechanisms, and disseminate information about training opportunities, such as those offered through the NCI’s Center to Reduce Cancer Health Disparities [e.g., career development awards; diversity supplements; cross-national initiatives such as the Geographic Management of Cancer Health Disparities Program (GMaP), Partnerships to Advance Cancer Health Equity (PACHE)]. Cancer disparities investigators who serve as leaders of training grants should actively recruit and support BIPOC trainees and provide opportunities for meaningful growth in mentored research, career development, and leadership to help reverse the negative effects of structural racism and racial battle fatigue on the success of BIPOC trainees in research and at predominantly White-serving institutions.

Through administrative and service activities, cancer disparities researchers can also promote institutional-level change to address the adverse effects of structural racism and support BIPOC scholars. Examples include advocacy and leadership in the implementation of strategic recruitment and hiring practices, which encourage BIPOC applicants, facilitation of research collaborations which include BIPOC, as well as openly discussing requirements and strategies for career advancement. In addition, faculty who identify as BIPOC frequently undertake additional service responsibilities related to issues of diversity, equity, and inclusion, and these faculty are also often in high demand for mentoring BIPOC students. In cases where unique experiences and perspectives cannot be replaced by other faculty members, administrative leaders should advocate that service requirements, assignments, and expectations be distributed more equitably across all faculty members to account for underrepresented faculty who engage in this unacknowledged additional labor.

## Call to Action #3: Recognize and Support Community Resilience/Actions to Address Structural Racism

Despite marginalization, BIPOC communities demonstrate resilience and have a rich, ongoing history of activism to raise awareness of and reduce the impact of structural racism. First, BIPOC communities focus on enhancing collective awareness about structural racism among community members and allies; disrupting the daily routines of cities, states, and the country at large that reflect structural racism; and motivating responsiveness of politicians/civic leaders to enable holistic antiracist policy change. Well-known tactics used to confront distal mechanisms of racism include marches and protests, which may be implemented due to widely publicized tragedies against BIPOC (e.g., murders of George Floyd and Breonna Taylor) or, *more commonly*, in response to the cumulative trauma these communities experience (e.g., persistent police brutality). Second, BIPOC historic (e.g., Black Panther Party, Young Lords) and contemporary groups (e.g., For the Breast of Us, PINK 4 the City, Touch the Black Breast Cancer Alliance) have employed strategies that help supplement the inequitable distribution of resources across various domains within society that result in cancer inequities (e.g., lack of access to healthy foods; lack of access to quality cancer care; medical apartheid). These efforts, often led by BIPOC leaders and stakeholders personally affected by cancer, highlight persistent inequities despite decades of attention to cancer as a societal problem.

Academic partners seeking to work with BIPOC populations to reduce cancer health disparities must be aware of and recognize BIPOC communities’ actions to effectively support and engage with these communities in the process of dismantling systemic oppression and balancing power differentials. Indeed, given the inadequacy of “colorblind” cancer prevention and control efforts, engagement of BIPOC communities—who, as abovementioned, may already be leading efforts relevant to cancer inequities—is critical to address their unique, multifaceted needs and eliminate persistent inequities. Toward that goal, researchers can support community efforts by attending protests; donating time, funding, and/or other resources to community groups/organizations confronting structural and other forms of racism; and engaging in political advocacy at local, state, and national levels. Academic partners can further lend their voices and resources to support initiatives to: (i) eliminate inequitable exposure to cancer risk factors (e.g., toxic waste sites); (ii) enhance structural and systemic efforts to mitigate exposure to risk factors (e.g., comprehensive marketing bans for tobacco, alcohol, etc.); and (iii) increase equitable access to high-quality protective factors (e.g., high-quality food environments; high-quality cancer care).

## Conclusion

This paper highlights how social, economic, and political injustices provide mechanisms through which structural racism contributes to cancer inequities. Further, we highlight ways in which the effects of structural racism on cancer disparities are magnified during the COVID-19 pandemic. Future research is necessary to provide more in-depth explorations of cancer and COVID-19 disparities within specific racial/ethnic groups, including assessing differences in structural racism and its consequences. Achieving cancer-related health equity requires intentional efforts to identify, understand, and address structural racism. Cancer researchers are called upon to appropriately identify structural racism within all aspects of scientific research; leverage our power and resources to dismantle structural racism within our academic institutions through teaching, mentoring, and service activities; and support community actions to address structural racism at the local, state, and national levels. Investigators may be required to seek additional training and build collaborations to develop and/or harness the expertise necessary to employ recommended strategies to address structural racism as it relates to cancer-related health disparities. Nonetheless, these approaches challenge sociocultural justifications for structural racism (e.g., individual blame, cultural generalizations) through acknowledgement and explicit discussions of societal forces. Moreover, they disrupt power imbalance and the multifaceted standards of practice across different systems (e.g., healthcare, education, criminal justice) through which structural racism is operationalized in society.
